# The Evolution of Monkeypox Vaccination Acceptance in Romania: A Comparative Analysis (2022–2025), Psychosocial Perceptions, and the Impact of Anti-Vaccination Rhetoric on Societal Security

**DOI:** 10.3390/bs15091175

**Published:** 2025-08-29

**Authors:** Cătălin Peptan, Flavius Cristian Mărcău, Olivia-Roxana Alecsoiu, Dragos Mihai Panagoret, Marian Emanuel Cojoaca, Alina Magdalena Musetescu, Genu Alexandru Căruntu, Alina Georgiana Holt, Ramona Mihaela Nedelcuță, Victor Gheorman

**Affiliations:** 1 Faculty of Educational Sciences, Law and Public Administration, “Constantin Brâncuși” University of Târgu Jiu, 210185 Târgu Jiu, Romania; catalin.peptan@e-ucb.ro (C.P.); flavius.marcau@e-ucb.ro (F.C.M.); olivia.alecsoiu@e-ucb.ro (O.-R.A.); alina.holt@e-ucb.ro (A.G.H.); 2 Faculty of Science and Engineering Alexandria—Valahia University of Târgoviște, 140003 Alexandria, Romania; dragos.panagoret@valahia.ro; 3 National Health Insurance House (CNAS), Titu Maiorescu University, 040051 Bucharest, Romania; marian.cojoaca@utm.ro; 4 Victor Babeș Hospital for Infectious and Tropical Diseases, 030303 Bucharest, Romania; 5 Faculty of Medicine, Titu Maiorescu University, 040051 Bucharest, Romania; 6 Faculty of Economics, “Constantin Brâncuși” University of Târgu Jiu, 210185 Târgu Jiu, Romania; genu.caruntu@e-ucb.ro; 7 Faculty of Medicine, University of Medicine and Pharmacy of Craiova, 200349 Craiova, Romania; ramona.nedelcuta@umfcv.ro; 8 Department of Psychiatry, University of Medicine and Pharmacy of Craiova, 200349 Craiova, Romania; victor.gheorman@umfcv.ro; 9 Department of Psychiatry I, Craiova Clinical Neuropsychiatry Hospital, 200473 Craiova, Romania

**Keywords:** monkeypox, vaccine, hesitancy, societal security, fake news, Romania

## Abstract

This study examines the evolution of willingness to accept the monkeypox (Mpox) vaccine in Romania between 2022 and 2025. It explores key sociodemographic and behavioral predictors of vaccine acceptance and investigates how public perceptions—particularly concerning disease severity and conspiracy beliefs—have shifted across two independent cross-sectional samples. Two nationally distributed surveys were conducted in July 2022 (n = 820) and January–February 2025 (n = 1029), targeting Romanian residents aged 18 and above. Data were analyzed using descriptive statistics, Chi-square tests, Kolmogorov–Smirnov tests, and a Random Forest classification model to assess the relative importance of predictors of vaccine acceptance. Between 2022 and 2025, vaccine acceptance increased modestly, particularly among individuals aged 36–65 and those with prior experience of voluntary or COVID-19 vaccination. Random Forest analysis identified behavioral factors as the strongest predictors of acceptance in both years, while the influence of education and gender varied over time. Belief in conspiracy theories slightly declined and lost predictive relevance by 2025. Perceptions of pandemic potential and fear of infection also decreased, suggesting reduced risk salience and possible pandemic fatigue. Despite a slight upward trend, overall Mpox vaccine acceptance in Romania remains among the lowest in Europe. These findings highlight the need for targeted public health communication, particularly toward skeptical or demographically vulnerable groups. Prior vaccination behavior emerged as a key driver of acceptance, indicating that trust-building strategies should capitalize on existing pro-vaccination habits. Future research should adopt qualitative and longitudinal approaches to better capture the evolving psychosocial dynamics of vaccine hesitancy.

## 1. Introduction

The resurgence of the monkeypox virus (Mpxv) in Europe, in the aftermath of the COVID-19 pandemic, has reignited concerns about public health preparedness, societal resilience, and the effectiveness of vaccination strategies (Peptan et al., 2022). During the 2022 outbreak, over 20,000 infections were reported in the European region alone (European Commission, 2019, prompting the World Health Organization (WHO) to declare a global public health emergency (Plummer, 2022). The situation escalated further in 2024, when a new, highly transmissible strain—Mpox clade Ib—was identified in the Democratic Republic of Congo and quickly spread to neighboring African countries. WHO subsequently issued another public health emergency of international concern (Ndembi et al., 2025). Isolated cases of this variant were later detected in several European countries, including Sweden, Germany, France, Belgium, Ireland, and the United Kingdom, although the overall risk of infection for the European population remained low (European Centre for Disease Prevention and Control, 2025).

Romania reported its first confirmed Mpox case on 13 June 2022. As of 15 February 2025, only 30 infections had been recorded nationally—all in men aged 24 to 51, with no severe complications (Graphs.ro, 2025). No cases of the Mpox clade Ib variant have been reported in Romania to date; however, the risk of introduction remains, given the country’s regional mobility and increasing number of travelers to Western Europe.

Despite the relatively low epidemiological impact in Romania, understanding public attitudes toward Mpox vaccination is essential—especially in light of the recent experience with COVID-19. The global vaccination campaign during the pandemic was accompanied by an unprecedented wave of skepticism, resistance, and misinformation, leading WHO to classify vaccine hesitancy as one of the top ten threats to global health (Lounis & Riad, 2023). In Romania, this phenomenon was particularly pronounced, with public discourse shaped by misinformation, anti-vaccination narratives, and low institutional trust (Dobre, 2021; Bârgăoanu, 2021; Voinea et al., 2023; Blănaru & Vaida, 2024; Tompea, 2022; Stoica, 2024).

Vaccination remains the most effective method for controlling the spread of viral diseases (European Commission, 2019; Peptan & Peptan, 2021). In the case of Mpox, both primary pre-exposure vaccination (PPV) and post-exposure preventive vaccination (PEPV) are recommended for individuals at increased risk of infection or severe disease (Sulaiman et al., 2024; European Centre for Disease Prevention and Control, 2025). However, knowledge gaps and low awareness regarding Mpox vaccination persist—not only among the general population but also among healthcare professionals (Wang et al., 2024; Tanashat et al., 2024; Kanyo et al., 2021). Recent studies report a global Mpox vaccine acceptance rate of approximately 65%, with significantly higher acceptance observed among individuals with higher education levels, prior COVID-19 or influenza vaccination, and those from vulnerable groups such as people living with HIV or members of the LGBTQ+ community (Borcak et al., 2024; Indiastari et al., 2024; Moawad et al., 2023; Lounis et al., 2024). In contrast, lower acceptance rates are consistently reported among individuals with lower education levels and those expressing traditionalist views on sexuality and gender identity.

Notably, recent research has demonstrated that vaccine hesitancy is not confined to socioeconomically disadvantaged groups. Individuals with higher education and income may also express skepticism—not necessarily due to misinformation, but as a result of ideological orientations such as healthism. This framework emphasizes personal responsibility for health, distrust of pharmaceutical interventions, and a preference for “natural” immunity, all of which can foster resistance to institutional vaccination campaigns. Healthism helps to explain why some well-informed individuals may reject vaccination on the basis of self-regulation, autonomy, and individualized health practices (Kirbiš, 2023).

In Romania, Mpox vaccine acceptance remains substantially lower than the European average. Several factors contribute to this trend, including ideological polarization, limited health literacy, and the widespread dissemination of conspiracy theories (Vâlcea, 2023). This reluctance is exacerbated by misleading public discourse and radical skepticism, both of which intensified during the COVID-19 crisis. Furthermore, despite advances in vaccine development—such as mRNA platforms and antibody-based immunotherapies—no Mpox-specific vaccine is currently available on the commercial market (Dutta et al., 2025; Singh et al., 2025; Halder et al., 2025). Consequently, prevention through public awareness and behavior change remains a strategic priority.

Academic interest in the social dimensions of Mpox has grown considerably, particularly regarding behavioral factors, misinformation, and societal resilience (Dascălu et al., 2021; Jain et al., 2022; Peptan & Mărcău, 2024; Rocha et al., 2021). Moreover, health is increasingly viewed not only as a medical issue but also as a pillar of national security. According to the Copenhagen School’s framework of societal security (Tompea, 2022), public health is considered essential for maintaining societal stability. In this study, we operationalize societal security through three measurable dimensions: (1) public trust in health institutions and vaccines, (2) risk perception related to emerging epidemics, (3) resilience to disinformation and conspiracy narratives. These dimensions reflect the core mechanisms identified by the Copenhagen School as essential to preserving social cohesion under threat. A weakening of these pillars—manifested through institutional distrust, pandemic fatigue, and susceptibility to fake news—is interpreted as a form of societal insecurity, with direct implications for public health planning. In Romania, this principle is enshrined in the national defense strategy, which identifies healthcare as a strategic sector through provisions for health education, access to medical services, and resource allocation (Stoica, 2024; Ordeanu, 2018; Tiberiu, 2019).

In this context, the present research examines how Romanians perceive the risk of Mpox and how these perceptions shape their willingness to vaccinate. Building on insights from the COVID-19 experience, the study argues that attitudes toward emerging epidemics are determined not only by epidemiological data, but also by psychological fatigue, behavioral memory, and the persistence of conspiracy narratives. Accordingly, we advance three guiding hypotheses: that reduced perception of Mpox as a pandemic threat and declining fear of infection are likely to lower vaccine acceptance (H1); that sociodemographic and behavioral factors—particularly prior vaccination experience—continue to structure willingness to vaccinate (H2); and that conspiracy beliefs, while still present, exert only a marginal influence on vaccination intent in the Romanian context (H3). By testing these assumptions across two waves of data collection (2022 and 2025), the study seeks to provide a nuanced understanding of the cultural and institutional dynamics that shape vaccine hesitancy and public health resilience.

## 2. Materials and Methods

### 2.1. Participants

The study was based on data collected via an online questionnaire administered between 10 January and 10 February 2025, targeting individuals aged 18 and above residing in Romania. No identifying information was gathered. Participation was voluntary, anonymous, and uncompensated. Respondents were informed about the authors’ institutional affiliations, the funding source of the study, and that the data would be used exclusively for scientific purposes.

The dataset collected in July 2022 had been previously subjected to descriptive analysis in a separate publication (Peptan et al., 2022). The present manuscript expands upon that work by incorporating newly collected data from 2025, formulating three comparative hypotheses, and applying inferential statistical methods to identify predictive patterns and behavioral dynamics over time. Notably, the 2022 publication did not include any inferential or comparative analyses.

### 2.2. Procedure

The questionnaire was developed using the Google Forms platform and distributed via social media platforms through a shared link. The aim was to reach participants across all geographic regions of Romania and ensure a diverse sociodemographic profile. Efforts were made to distribute the survey link within communities similar to those targeted in the 2022 study, thereby minimizing significant differences in the sample composition.

Access to the questionnaire was restricted to individuals who selected “Yes” in response to a screening question confirming that they were at least 18 years old and resided in Romania.

To maintain methodological consistency between the 2022 and 2025 studies, identical distribution methods and sampling approaches were used. This ensured the comparability of results across both waves and enabled a clearer identification of trends and shifts in public perceptions.

### 2.3. Measurements

The questionnaire included a set of questions that allowed for data collection in three distinct sections: (1) sociodemographic data of the study participants (age, gender, place of residence, and educational background); (2) perceptions regarding vaccine acceptance, including views on mandatory vaccines in Romania, acceptance of optional vaccines, trust in Mpox vaccination, and perspectives on the evolution of Mpxv; (3) opinions on misinformation, specifically fake news circulating in the public sphere related to Mpxv.

### 2.4. Statistical Data Analysis

The statistical analysis was conducted in Google Colab (2025 version) using Python-based packages (Python 3.10.12, Pandas 2.2.2, NumPy 1.26.4, SciPy 1.13.1, Matplotlib 3.9.0, and scikit-learn 1.5.0), complemented by Microsoft Excel 2021 (Office 365 version) for initial preprocessing. The raw data collected through Google Forms were exported into Excel and then uploaded into Colab, where all computations and visualizations were performed. The key sociodemographic variables included age, gender, place of residence, and education level.

To compare response patterns between the 2022 and 2025 datasets, several statistical tests were used: Chi-square tests assessed associations between categorical variables (e.g., gender and vaccine acceptance); Kolmogorov–Smirnov tests were applied to ordinal items with more than two response levels; independent samples t-tests examined mean differences across demographic groups.

To identify predictors of vaccine acceptance and belief in Mpox-related narratives, ordinal logistic regression models were constructed. These models estimated the influence of variables such as age, gender, education, and residence on vaccine hesitancy across the two time points.

Additionally, a Random Forest classification model was employed to assess variable importance in predicting willingness to vaccinate. This non-parametric, machine-learning approach provided a data-driven analysis of the factors most strongly associated with vaccination intent—highlighting the significance of prior vaccination behavior, demographic characteristics, and susceptibility to conspiracy beliefs.

Together, these analyses provided a robust, multidimensional understanding of evolving public attitudes toward vaccination and health-related behavior in Romania between 2022 and 2025.

## 3. Results

### 3.1. Sociodemographic Data

The study is based on a comparative analysis of the results obtained from online surveys conducted between 1 and 31 July 2022 (820 respondents) (Peptan et al., 2022) and 10 January and 10 February 2025 (1029 respondents), targeting individuals aged 18 and above residing in Romania. The sociodemographic data of the participants are presented in Table 1. Compared to the initial study (Peptan et al., 2022), the age groups of respondents were restructured to provide a clearer synthesis of the results.

The following aspects stand out: the lower proportion (32.95%) of young respondents (18–35 years old) who participated in the 2025 study, compared to the proportion (53.40%) of those who participated in the 2022 study; the higher proportion (40.04%) of adult respondents (36–45 years old) who participated in the 2025 study, compared to the proportion (21.25%) of those who participated in the 2022 study; small differences between the proportions of male respondents (54.62% in 2025, 50.38% in 2022) and female respondents (45.38% in 2025, 49.62% in 2022) and those residing in urban areas (79.30% in 2025, 72.44% in 2022) and rural areas (20.70% in 2025, 27.56% in 2022), as well as those with pre-university education (19.29% in 2025, 21.03% in 2022) and university education (82.02% in 2025, 78.97% in 2022). Except for the variable of respondents’ age, the structure of the surveyed samples in 2022 and 2025 remains similar.

### 3.2. Public Perception and Pandemic Belief (H1)

The comparison of data collected in 2022 and 2025 indicates a general downward trend in public perception of pandemic risk and fear of Mpox infection, as shown in Table 2.

Belief in Mpox’s pandemic potential (Q1) and fear of infection (Q2) declined significantly between 2022 and 2025—from 21.3% to 11.9% and from 30.9% to 18.1%, respectively. These trends were statistically significant (*p* < 0.01, Chi-square and Kolmogorov–Smirnov tests) and evident across demographic groups, although the intensity varied. Notably, concern among respondents aged over 66 dropped to negligible levels, indicating a perceived irrelevance of the threat within this cohort.

**Figure 1:**
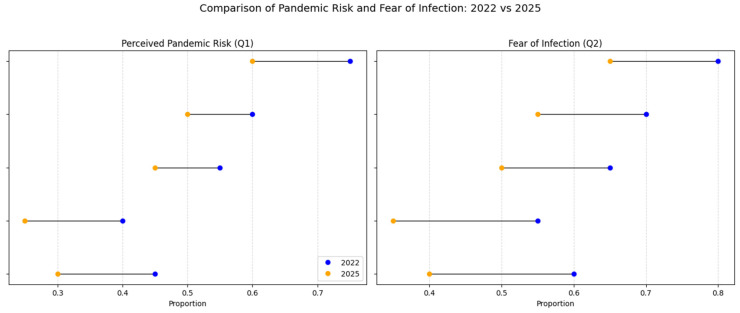
Change in pandemic risk and fear of infection perceptions (2022 vs. 2025).

Figure 1 visually illustrates these trends, showing consistent declines across all key subgroups, including youth, urban residents, and individuals with higher education. The contraction of perceived risk was especially pronounced among groups that previously reported high levels of concern—such as those vaccinated against COVID-19—suggesting a broader affective disengagement.

These findings support Hypothesis H1 and point to a wider psychological detachment, likely driven by pandemic fatigue and the low incidence of Mpox at the national level. While the decline in perceived risk is understandable, it may undermine proactive health behaviors. Even amid a slight increase in the stated willingness to vaccinate, the data reveal a disconnect between recognition of the threat and readiness to act. This underscores the need for sustained, targeted public health communication capable of maintaining vigilance in low-incidence settings.

### 3.3. Sociodemographic Predictors of Vaccine Acceptance (H2)

To assess the predictive influence of sociodemographic and behavioral variables on the acceptance of Mpox vaccination (Q3: “Would you agree to get vaccinated against Mpox?”), we applied a Random Forest algorithm to two independent datasets from 2022 and 2025. In both cases, the dependent variable was recoded into a binary format to reflect a clear distinction between acceptance (1) and refusal (0); neutral responses were excluded from the 2025 dataset.

**Figure 2:**
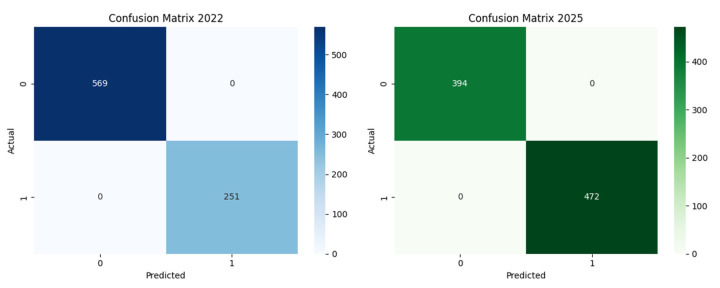
Confusion matrices showing the Random Forest model performance for the 2022 and 2025 datasets.

The trained models demonstrated exceptionally high performance, achieving 100% classification accuracy in both cases. This indicates a perfect ability to distinguish between respondents who accepted and those who refused vaccination. Figure 2 presents the confusion matrices corresponding to the two models, illustrating their classification accuracy.

To identify the most influential predictors in each model, we analyzed the variable importance scores generated by the algorithm. Table 3 summarize these results, showing the relative importance of each predictor across both datasets.

As shown above, uptake of optional vaccines (Q4) emerged as the strongest predictor of Mpox vaccine acceptance in both waves of the study, indicating a stable relationship between prior voluntary vaccination behavior and current decision-making. In 2025, the predictive importance of COVID-19 vaccination (Q5) increased significantly, potentially reflecting the influence of pandemic-related experiences on attitudes toward new vaccines.

The remaining sociodemographic variables—gender, age, education, and place of residence—had limited predictive power in both years and showed no substantial variation over time.

**Figure 3:**
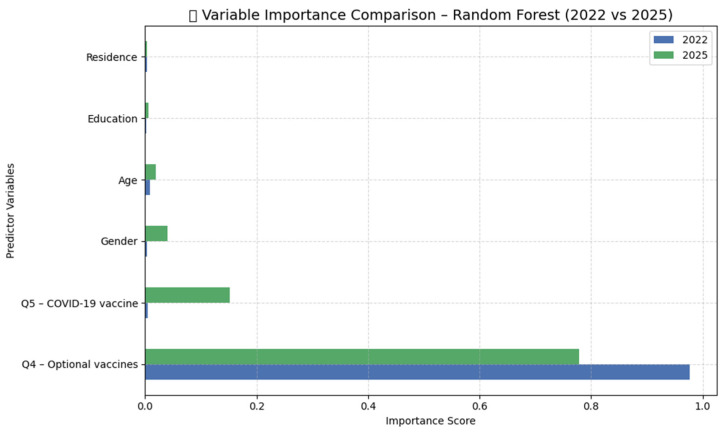
Performance of Random Forest models in classifying vaccine acceptance responses: confusion matrices for 2022 and 2025.

To facilitate a more intuitive comparison, Figure 3 presents the relative importance of all predictors across the two datasets.

Table 4 summarizes the results of logistic regression models applied to each of the five conspiracy-related items (MS1–MS5), treated as dependent variables, in relation to a set of sociodemographic predictors (age, gender, education, and place of residence), calculated separately for the years 2022 and 2025. The aim of these analyses was to identify population groups with a significantly higher likelihood of endorsing conspiratorial explanations regarding Mpxv.

Prior to conducting the regression analyses, an exploratory factor analysis (EFA) was performed to assess the factorial validity of the five-item scale used to measure conspiracy beliefs (MS1–MS5). The EFA confirmed the unidimensionality of the construct across both waves of data. In 2022, the Kaiser–Meyer–Olkin (KMO) measure was 0.873, and Bartlett’s test of sphericity was significant (χ2 = 3244.82, p < 0.001), indicating that the data were suitable for factor analysis. All five items loaded strongly onto a single factor, with standardized loadings ranging from 0.85 to 0.91.

Similarly, in 2025, the KMO value was 0.885, and Bartlett’s test remained highly significant (χ2 = 6757.44, p < 0.001). Factor loadings for all five items exceeded 0.90, further supporting a stable and coherent unifactorial structure across both waves. These results validate the conceptual integrity of the MS1–MS5 scale and justify its use as a latent indicator of conspiratorial thinking related to Mpxv.

**Figure 4:**
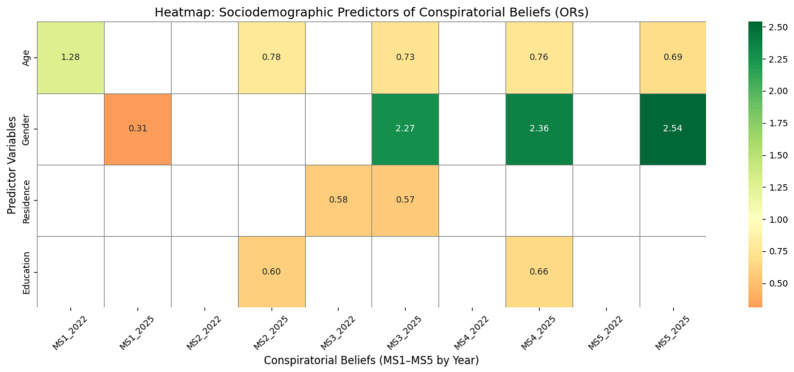
Heatmap of sociodemographic predictors of conspiratorial beliefs in 2022 and 2025 (odds ratios).

The logistic regression analyses in Table 4, complemented by the heatmap in Figure 4, reveal notable and evolving sociodemographic patterns in the endorsement of conspiratorial beliefs surrounding Mpox.

Age, which was not significant in 2022, emerged in 2025 as a positive predictor for belief in the reality of Mpox (MS1; OR = 1.28, *p* < 0.01), while female gender appears associated with lower agreement (OR = 0.31, *p* < 0.01), suggesting the formation of a gendered perception gap regarding official narratives.

Education and residential setting consistently influence conspiratorial thinking. In 2022, lower education levels predicted belief in the idea that Mpox was created by elites (MS2; OR = 0.58, *p* < 0.05), a pattern strengthened by 2025 (OR = 0.60, *p* < 0.05). Rural residence and male gender also become significant predictors in 2025 for the same belief, with particularly high odds among men (OR = 2.27, *p* < 0.01) and those living in rural areas (OR = 0.73, *p* < 0.05).

Belief in the idea that Mpox is a manipulation tool (MS3) is also shaped by age and gender. In 2022, younger age and rural residence were significant factors (ORs = 0.58 and 1.17, respectively), while in 2025, male respondents were significantly more likely to hold this belief (OR = 2.36, *p* < 0.01).

The view that Mpox is a vaccine for population reduction (MS4) remains relatively stable across years, with rural residence (2022 OR = 0.49, 2025 OR = 0.76) and lower education (2022 OR = 0.49, 2025 OR = 0.66) consistently associated with higher endorsement. Notably, male gender became a significant factor in 2025 (OR = 2.54, *p* < 0.01), suggesting a deepening of conspiratorial framing among men.

Lastly, belief in the population control theory (MS5) remains strongly linked to rural residency (2022 OR = 0.57; 2025 OR = 0.69) and lower education (2025 OR = 0.66), though the magnitude of these associations slightly weakens over time.

These findings reinforce Hypothesis H2 and underline that conspiratorial belief systems remain disproportionately concentrated among specific sociodemographic groups—particularly rural men with lower educational attainment. While some associations remained stable across years, the 2025 data suggest not a general demographic shift, but rather an intensification of conspiratorial alignment among younger, rural males, pointing to the resilience—and perhaps radicalization—of belief clusters in specific social segments.

Table 5 summarizes the results of logistic regression models assessing the influence of conspiratorial beliefs (MS1–MS5) on willingness to vaccinate against Mpox (Q3), across two temporal waves: 2022 and 2025.

**Figure 5:**
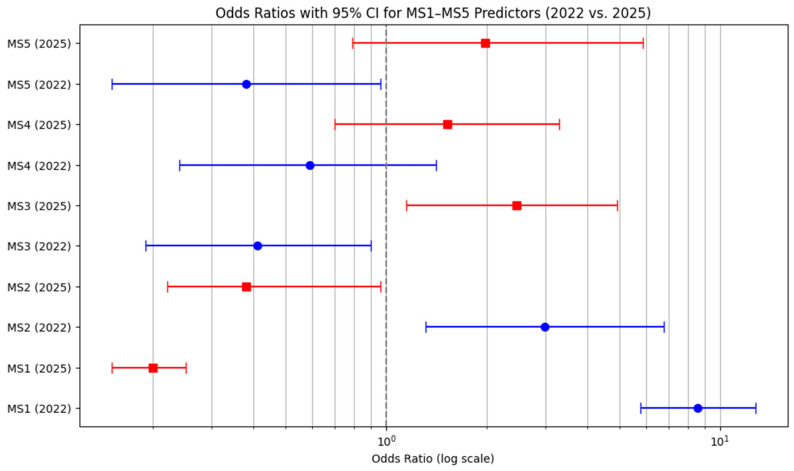
Odds ratios (OR) with 95% confidence intervals for MS1–MS5 predictors of Mpox vaccine acceptance (2022 vs. 2025).

The logistic regression results summarized in Table 5 and visualized in Figure 5 highlight important changes in the influence of conspiracy beliefs on vaccination intent between 2022 and 2025.

The item MS1 (“Mpox is real”) shows the most striking reversal: in 2022, agreement with this statement was a strong positive predictor of willingness to vaccinate (OR = 8.59, CI: 5.77–12.78, *p* < 0.01), whereas in 2025 it became a significant negative predictor (OR = 0.20, CI: 0.15–0.25, *p* < 0.01). This dramatic shift suggests a substantial reconfiguration in how such beliefs relate to behavior, possibly reflecting evolving public attitudes or broader changes in the informational landscape.

For MS2 (“Mpox was created by elites”), a significant positive association with vaccine acceptance observed in 2022 (OR = 2.98, CI: 1.31–6.79, *p* < 0.01) disappeared by 2025 (OR = 0.38, CI: 0.22–0.96, *p* > 0.05), indicating a decline in its predictive value.

A similar pattern is seen with MS3 (“Mpox is a manipulation tool”), which shifted from a significant negative predictor in 2022 (OR = 0.41, CI: 0.19–0.90, *p* < 0.05) to a strong positive predictor in 2025 (OR = 2.45, CI: 1.15–4.91, *p* < 0.01), suggesting a redefinition of the meaning or connotation attached to such narratives.

Interestingly, MS4 (“Mpox vaccines are used for population reduction”) showed a positive association with vaccine acceptance in 2025; however, the effect was not statistically significant (*p* > 0.05), indicating that while some respondents may reconcile political skepticism with willingness to vaccinate, the evidence remains inconclusive.

Finally, MS5 (“Mpox is a population control mechanism”) had a significant negative effect in 2022 (OR = 0.38, CI: 0.15–0.96, *p* < 0.05) but became non-significant by 2025 (OR = 1.98, CI: 0.79–5.87, *p* > 0.05), further suggesting a decline in the behavioral influence of extreme conspiracy narratives.

Overall, these findings offer partial support for Hypothesis H3. While conspiracy beliefs were strongly associated with vaccine hesitancy in 2022, their predictive power had largely diminished by 2025. This evolution may reflect growing public resilience to disinformation, potentially reinforced by prior vaccination experience and more effective public health communication.

### 3.4. Summary Diagram and Key Patterns

To visually consolidate the empirical findings and support the validation of the study’s hypotheses (H1–H3), we propose a simplified conceptual model of the relationships between key predictors and vaccination willingness.

**Figure 6:**
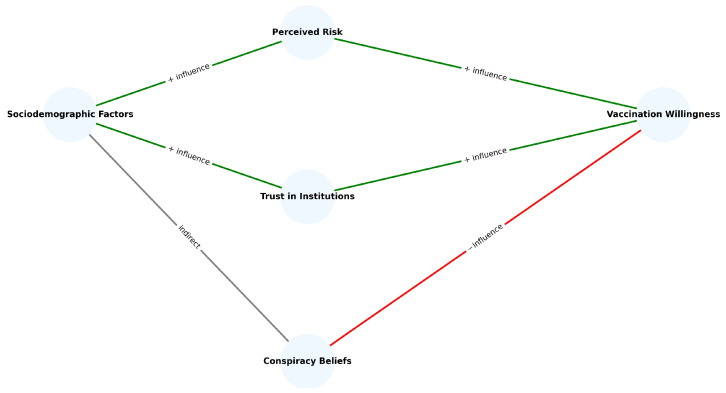
Predictive model of vaccination willingness.

The diagram presented in Figure 6 illustrates the conceptual structure derived from the statistical analyses conducted in this study. Solid arrows indicate statistically significant predictive relationships identified through the regression models. The model consists of three levels: (1) sociodemographic factors (age, gender, education, residence), (2) cognitive–affective mediators (perceived risk, trust in health institutions, belief in conspiracy theories), (3) behavioral outcome (willingness to accept Mpox vaccination). This structure reflects the empirical validation of Hypotheses H1–H3 and offers a synthesized visualization of the key factors shaping vaccine-related decisions in Romania between 2022 and 2025.

Taken together, the results across all statistical models and time points provide a consistent and multidimensional comparison of responses collected in the two survey waves. As shown in Section 3.1, concern about Mpox—both in terms of its perceived pandemic potential (Q1) and fear of personal infection (Q2)—was lower in 2025 than in 2022 across nearly all sociodemographic categories, supporting Hypothesis H1. This decline may reflect reduced perceptions of threat severity and the broader effects of pandemic fatigue.

In terms of vaccination willingness (Q3), the 2025 sample reported a modest but statistically significant increase in affirmative responses, particularly among individuals aged 36–65, urban residents, and those with a history of voluntary or COVID-19 vaccination. These findings support Hypothesis H2 and indicate that these variables were more strongly associated with vaccination intent in 2025 compared to 2022.

The results related to belief in conspiracy theories about Mpox (MS1–MS5) also reveal important differences between the two survey waves. While agreement with such statements was negatively associated with vaccine acceptance in 2022, these correlations weakened considerably by 2025, with some items losing statistical significance altogether. As detailed in Section 3.3 and Table 4, these patterns support Hypothesis H3 and may reflect the cumulative impact of institutional communication and broader public exposure to health-related messaging during the post-pandemic period.

## 4. Discussion

### 4.1. Pandemic Perception and Risk Fatigue (H1)

The comparison between the 2022 and 2025 datasets confirms Hypothesis H1: diminished belief in Mpox’s pandemic potential and reduced fear of infection are associated with lower overall vaccine acceptance. Statistically significant changes in pandemic perception and infection-related anxiety were observed between the two time points. Specifically, belief in the possibility of an Mpox pandemic (Q1) declined from 21.3% in 2022 to 11.9% in 2025, a difference confirmed by Chi-square analysis (χ^2^ = 298.93, *p* < 0.01). Among the most relevant sociodemographic predictors were place of residence and gender, followed by education and age.

Similarly, fear of infection (Q2) decreased from 30.9% to 18.1%, with the Kolmogorov–Smirnov test (KS = 0.257, *p* < 0.01) confirming significant distributional differences between the two years. The steepest declines were observed among urban and younger respondents, who reported the lowest levels of perceived vulnerability in 2025.

Several factors likely contributed to this shift. First, the very low number of reported Mpox cases in Romania (30 by early 2025, all mild) likely reduced the sense of urgency. Second, pandemic fatigue—a form of psychological disengagement following prolonged exposure to health crises such as COVID-19—appears to have diminished public responsiveness to emerging infectious threats. Third, increased public familiarity with zoonotic viruses and the generally neutral tone of institutional communication may have contributed to a more rational, measured perception of risk (Tompea, 2022; Ungureanu, 2022; Badea & Mișacă, 2021).

The declining media visibility of Mpox, especially when compared to the saturation coverage of COVID-19, likely reinforced this trend. Both WHO and ECDC guidelines emphasize that risk perception is shaped by narrative framing, message frequency, and the visibility of institutional actors (Emanuel et al., 2023).

These developments are not unique to Romania. Similar patterns have been observed in other Central and Eastern European countries, where weaker institutional trust and fragmented public health messaging often contribute to a rapid erosion in perceived urgency—unless reinforced by sustained outbreaks or targeted communication efforts (Trifunović, 2022; Cadeddu, 2023; Székely et al., 2024). By contrast, countries such as Germany and France have maintained higher levels of vaccine acceptance, supported by consistent messaging and strong institutional credibility (SHARE-ERIC, 2021; Jentzsch et al., 2022).

In summary, the perception of Mpox as a pandemic threat declined significantly between the two waves. The reduction in fear and urgency—particularly among older and more informed respondents—suggests a broader normalization of epidemic risk. While this trend is understandable in a post-COVID context, it presents challenges for future public health interventions. These findings confirm Hypothesis H1 and underscore the importance of sustained, credible risk communication in maintaining public readiness.

### 4.2. Sociodemographic and Behavioral Predictors of Vaccine Acceptance (H2)

Hypothesis H2 proposed that vaccine acceptance is shaped by key sociodemographic factors such as age, gender, place of residence, and education level. Findings from both waves of data collection support this assumption, revealing both continuity and change in how these variables influence willingness to vaccinate against Mpox.

To assess the relative importance of these predictors, a Random Forest classification model was trained on each dataset using Google Colab. The models demonstrated excellent performance, correctly classifying all cases based on the included variables. In both 2022 and 2025, behavioral factors emerged as the most influential predictors. Prior voluntary vaccination (Q4) and COVID-19 vaccination (Q5) consistently ranked as the top contributors to vaccine acceptance. These results align with the existing literature highlighting the predictive role of previous vaccination behavior in shaping future uptake intentions (Gherheș et al., 2023; Matei et al., 2021; Mărcău et al., 2022; Sandu, 2023; Lawrence, 2024).

In 2022, willingness to vaccinate was modest, with the highest proportions observed among women (39.5%) and individuals aged 26–35 (39.6%). Sociodemographic factors such as urban residence, gender, and age exhibited some predictive power, though their influence was secondary to behavioral factors. Urban respondents were more likely to accept the Mpox vaccine, while older individuals and women showed slightly lower levels of acceptance.

By 2025, the profile of vaccine acceptance had become more consolidated. The overall acceptance rate increased from 41.57% to 45.87%, particularly among individuals aged 36–65. The Kolmogorov–Smirnov test confirmed significant shifts in response distributions (*p* < 0.01), especially within the 36–45 and 46–55 age groups. However, Random Forest analysis indicated that age and gender remained secondary predictors, while education level had minimal influence in both years. These findings are consistent with recent studies suggesting that education is no longer a consistent determinant of vaccine acceptance in post-pandemic contexts (Trifunović, 2022; Cadeddu, 2023; Székely et al., 2024).

Notably, COVID-19 vaccination emerged as the strongest single predictor of Mpox vaccine acceptance in 2025, reinforcing the idea that public trust and behavior are shaped by prior experiences with immunization campaigns. These findings validate Hypothesis H2 and align with international research emphasizing the importance of behavioral memory and institutional credibility in future vaccine uptake (Sandu, 2023; Lawrence, 2024).

The sociodemographic patterns observed in Romania mirror findings from other Central and Eastern European countries such as Bulgaria, Hungary, and Slovakia, where vaccine acceptance also tends to be lower among rural populations, women, and younger individuals. In contrast, studies from Western Europe report more consistent vaccine uptake across demographics, attributed to higher levels of institutional trust and stronger public health infrastructure (Trifunović, 2022; Cadeddu, 2023; Székely et al., 2024; SHARE-ERIC, 2021). For instance, in Germany and France, urban–rural disparities in vaccine behavior have been less pronounced, owing to proactive outreach campaigns and universal access to immunization services (Jentzsch et al., 2022). Moreover, the predictive value of prior COVID-19 vaccination on Mpox vaccine willingness has been documented in countries such as the United Kingdom and Canada, where public health messaging framed Mpox immunization as a continuation of civic responsibility (Gherheș et al., 2023; Matei et al., 2021).

These international parallels reinforce the notion that behavioral predictors—especially past vaccination behavior—can serve as robust levers for designing effective public health campaigns, even in settings where demographic barriers persist.

Overall, these findings strongly validate Hypothesis H2 and highlight the need for differentiated communication strategies. Efforts to increase vaccine acceptance should specifically target women, rural residents, and younger individuals, while also leveraging existing trust among those previously vaccinated. The persistence of gender-based disparities and the limited predictive power of education suggest that public health strategies must address underlying sociocultural attitudes and structural barriers to trust—not merely deficits in information (Emanuel et al., 2023; SHARE-ERIC, 2021).

Furthermore, recent scholarship emphasizes that vaccine hesitancy among highly educated individuals may not stem from a lack of knowledge, but rather from ideologically driven beliefs such as healthism—the conviction that health is primarily a matter of individual responsibility and that “natural” methods of prevention are superior to institutional medical solutions. Within this framework, vaccines may be viewed as unnecessary or even intrusive, especially by individuals who prioritize self-regulation, autonomy, and lifestyle-based immunity over collective immunization. These attitudes reflect a broader cultural orientation that distrusts government mandates and pharmaceutical authority, even when factual knowledge is present (Kirbiš, 2023).

By integrating this perspective, our findings regarding the inconsistent role of education in predicting vaccine acceptance can be better understood as reflecting ideological differentiation rather than cognitive deficit. This underscores the need for public health messaging that not only conveys accurate information, but also addresses deeper cultural narratives about health, autonomy, and trust.

### 4.3. The Role of Conspiracy Beliefs (H3)

Hypothesis H3 proposed that belief in Mpox among the Romanian population would only be weakly influenced by conspiracy theories (i.e., fake news), due to increased exposure to medical education and institutional communication. The findings partially confirm this hypothesis: while conspiracy beliefs persisted across both waves, their association with vaccination intent shifted considerably between 2022 and 2025.

Analysis of responses to items MS1–MS5 reveals a general decline in agreement with conspiracy-based narratives about the virus. Although agreement with MS1 (“Mpox is real”) remained relatively high, its predictive effect reversed dramatically—from a strong positive association with vaccination intent in 2022 to a significant negative one in 2025. This suggests a dissociation between disease recognition and protective behavior, reflecting acknowledgment of the disease’s existence even as perceived risk diminished. In contrast, agreement with more extreme claims—such as MS4 (“Mpox vaccines were created to reduce the world’s population”) and MS5 (“Mpox emerged because COVID-19 didn’t reduce the population enough”)—decreased in 2025, particularly among older and more educated individuals. These patterns suggest increased public resilience to speculative or sensationalist narratives.

Chi-square tests and independent samples t-tests confirm statistically significant differences across most items between 2022 and 2025, indicating a shift in conspiratorial perceptions. The most pronounced reductions were observed among urban respondents and individuals with higher levels of education, consistent with trends reported in other Central and Eastern European countries (Trifunović, 2022; Omojunikanbi, 2023; Sîrbu, 2020).

When tested as predictors of vaccination willingness (Q4), these items revealed a complex pattern. In 2022, some conspiracy-related beliefs were weakly negatively correlated with vaccination intent. By 2025, most of these associations had diminished or lost statistical significance. Notably, belief in MS3 and MS5—both referencing population control or biopolitical manipulation—had minimal predictive power in 2025, suggesting that such narratives lost traction in shaping vaccine behavior.

A surprising exception was MS4 (“Mpox vaccines for population reduction”), which in 2025 emerged as a positive predictor of vaccine acceptance. This paradox may reflect a shift in how some respondents rationalize global events: even when attributing political motives to vaccination campaigns, they may still perceive immunization as a valid personal health measure. This aligns with the concept of “conspiracy reframing”, in which distrust in institutions coexists with a pragmatic orientation toward individual health (Székely et al., 2024; Lawrence, 2024).

Logistic regression models further indicate that in 2025, age and gender were the strongest predictors of conspiracy belief endorsement, with younger males expressing higher levels of agreement. Unlike in 2022, education level had a reduced effect, suggesting a more complex attitudinal environment in which exposure to medical information and institutional communication may outweigh formal education (Cadeddu, 2023; Gherheș et al., 2023; Matei et al., 2021; Mărcău et al., 2022; Sandu, 2023).

Taken together, these findings suggest that conspiracy beliefs remain present in Romanian society but have a diminished relationship with preventive health behavior compared to 2022. This shift likely reflects the impact of institutional efforts to combat disinformation during and after the COVID-19 pandemic (Oeser et al., 2024; Hong et al., 2023; Mahameed et al., 2023). It also underscores the ongoing need for targeted health communication, particularly toward vulnerable groups more susceptible to misinformation.

Between 60% and 75% of respondents in Western European countries and Canada reported willingness to receive an Mpox vaccine—far exceeding the levels observed in Romania. For example, a comprehensive meta-analysis of 61 studies across 87 countries found that vaccine acceptance in the WHO European Region was 80.9% among LGBTQI+ individuals, 65.1% among people living with HIV, and 59.7% globally across all population groups (Sulaiman et al., 2024; León-Figueroa et al., 2024; Yappalparvi et al., 2025). Likewise, in France and Germany, surveys among high-risk populations (e.g., MSM using PrEP, healthcare workers) consistently reported vaccine acceptance rates above 65%, often surpassing 70%, attributed to strong institutional messaging and high disease awareness (European Centre for Disease Prevention and Control, 2025; Oeser et al., 2024).

In contrast, studies from Central and Eastern Europe—including Romania—reported significantly lower levels of vaccine acceptance. A Romanian study among life sciences students found a 48.5% acceptance rate, with high conspiracy belief scores strongly associated with refusal (Peptan et al., 2022). Broader surveys in neighboring countries mirrored this hesitancy: acceptance rates remained below 50%, despite public health messaging emphasizing elevated infection risk. These trends reflect persistent skepticism rooted in low institutional trust and weaker public health communication systems.

Looking beyond Europe, high acceptance rates among healthcare workers and general populations in Asia offer additional insight. In China, for example, 90.12% of healthcare workers surveyed expressed willingness to receive an Mpox vaccine, citing clear institutional protocols and a strong sense of professional duty (Hong et al., 2023). Similarly, in countries like Jordan and the United States, acceptance among healthcare professionals often exceeded 50%, depending on prior vaccination experience and perceived disease severity (Mahameed et al., 2023). These regional contrasts highlight the influence of institutional coherence and professional norms on vaccine uptake, beyond mere epidemiological risk.

Overall, the Romanian case fits within a broader regional pattern: compared to Western counterparts, countries in Eastern Europe—including Romania—continue to show lower Mpox vaccine acceptance. With levels below 40% across both waves, Romania ranks among the lowest in Europe. This divergence appears linked not only to sociodemographic factors but also to deeper structural issues, including low institutional trust, limited media literacy, and fragmented risk communication strategies. These findings reinforce the argument that vaccine hesitancy must be examined within its full structural, cultural, and institutional context (Mărcău et al., 2022; Omojunikanbi, 2023).

The relationship between conspiracy beliefs and vaccine hesitancy is not only an individual phenomenon—it is also shaped by macro-level contextual factors. As Lamot and Kirbiš (2024) demonstrate, country-level variables such as GDP per capita, perceived corruption, and dominant cultural dimensions (e.g., collectivism vs. individualism) can significantly moderate this association across Europe. Their cross-national analysis found that in countries with low institutional trust and high perceived corruption, conspiracy beliefs were more strongly linked to vaccine refusal. Romania—scoring high on collectivism and low on institutional trust in comparative indexes (e.g., Transparency International, Hofstede Insights)—illustrates this pattern. In our study, the strong predictive power of conspiracy-related items (MS2–MS5) may thus reflect not just personal belief systems, but broader societal mistrust rooted in a political culture shaped by opacity, paternalism, and fragmented authority. This underscores the need for culturally adapted public health messaging that goes beyond correcting misinformation and also addresses the systemic sources of skepticism (Lamot & Kirbiš, 2024).

In conclusion, Hypothesis H3 is partially supported: although conspiracy beliefs remained present in both samples, their association with vaccine intent was significantly weaker in 2025. This shift suggests that future public health communication strategies could prove effective.

### 4.4. Policy Implications and Future Research

The findings of this study carry important and actionable implications for public health strategy and policy implementation in Romania and in comparable socio-political contexts.

First, the significant decline in perceived pandemic risk and fear of infection between 2022 and 2025—even amid rising global Mpox cases—suggests that public alertness cannot be sustained through emergency declarations alone. Health authorities must adopt sustained, adaptive communication strategies that account for psychological fatigue in post-pandemic societies. This includes rotating risk narratives, delivering emotionally resonant messages, and leveraging trusted spokespersons—such as local doctors and public health experts—to enhance message credibility. Special attention should be given to younger individuals and women, who consistently reported lower willingness to vaccinate across both waves (Yatsuya et al., 2022).

Second, the observed behavioral consistency among individuals previously vaccinated—either through COVID-19 campaigns or other optional programs—presents a key opportunity. National health campaigns should frame Mpox vaccination as a natural continuation of responsible health behavior. This could involve direct comparisons or testimonials from previously vaccinated individuals. Personalized messaging, such as SMS reminders referencing prior vaccination history, could reinforce this continuity narrative (Tian et al., 2024; Jung et al., 2024).

Third, although belief in conspiracy theories declined slightly, certain population groups—particularly rural residents, individuals with lower education levels, and men—remain especially vulnerable to misinformation. To reach these segments, community-based outreach initiatives should be implemented, involving trusted intermediaries such as family physicians, teachers, and religious leaders. These efforts should include face-to-face educational sessions, Q&A forums, and the distribution of visual materials that directly address and debunk common myths.

Fourth, public health institutions should integrate real-time digital tools to monitor public sentiment and dynamically adjust communication strategies. This includes using social listening platforms to identify emerging narratives, dashboards to track emotional responses such as fear or resistance, and A/B testing to evaluate message effectiveness before national rollouts. These approaches, already recommended by WHO and ECDC, would be particularly valuable in Romania, where public trust in institutions remains fragile and subject to fluctuation (Okutsu & Goromaru, 2024).

In summary, public health efforts must move beyond abstract information campaigns toward more tactically segmented and behaviorally grounded interventions. Future strategies should be personalized, locally anchored, and data-driven in order to address the complex mix of mistrust, fatigue, and fragmented public opinion identified in this study.

Building on the methodological limitations of this research—particularly its reliance on cross-sectional survey data—future studies should incorporate qualitative methods such as in-depth interviews or ethnographic approaches. These would allow for deeper exploration of how individuals navigate conflicting information, construct meaning, and rationalize vaccination decisions in shifting contexts. Additionally, longitudinal designs could provide valuable insight into how media exposure, institutional trust, and public health interventions interact over time to shape vaccine behavior.

Comparative research across Southeastern Europe would also be highly valuable. Such studies could illuminate shared cultural, economic, and political factors that shape vaccine hesitancy in the region. This is especially relevant in societies characterized by populist rhetoric, low institutional trust, and widespread misinformation. Findings from such comparative work could help to guide the development of culturally adaptive and politically resilient strategies to rebuild public confidence in science and health authorities.

## 5. Limitations of the Study

This study is subject to several limitations that must be acknowledged when interpreting the findings.

First and foremost, the sample is not representative of the general Romanian population, as both waves of data collection (2022 and 2025) relied exclusively on online surveys. While the sample sizes were substantial—820 respondents in 2022, and 1029 in 2025—the recruitment method resulted in a demographic skew, with an overrepresentation of urban and university-educated individuals. In 2025, for example, 79.3% of respondents resided in urban areas and 82% held tertiary education degrees. In contrast, national statistics indicate that only approximately 54.3% of Romanians live in urban settings and just 18.6% of adults aged 25–64 have completed higher education. This imbalance likely inflated overall vaccine acceptance rates, as prior research shows that urban and highly educated populations tend to have greater trust in institutions and respond more positively to public health messaging. Therefore, our results—particularly the moderately high levels of acceptance—should be interpreted with caution and are not fully generalizable to the broader Romanian population, especially to rural or socioeconomically disadvantaged groups. Although no weighting procedures were applied, future studies should consider post-stratification adjustments or quota sampling to enhance the robustness of survey-based estimates.

Second, the exclusive use of an online survey platform restricted participation to individuals with internet access, thereby excluding digitally marginalized groups such as older adults or residents of remote rural areas. Moreover, the voluntary and anonymous nature of the survey may have introduced self-selection bias: individuals with strong opinions or heightened interest in vaccination-related topics were more likely to complete the questionnaire, potentially skewing the sample and reducing its neutrality (Bachelet et al., 2022).

A further methodological concern involves the structural inconsistency between the 2022 and 2025 versions of item Q2. In 2025, a neutral response option was added to the Likert scale due to platform constraints. While a non-parametric comparison (Kolmogorov–Smirnov test) was used to assess distributional differences, we also conducted a sensitivity analysis by collapsing the 2025 responses into a binary variable to mirror the 2022 format. This allowed for a more direct temporal comparison, and the results remained robust after recoding. Nevertheless, strict comparability of perceived infection-related fear over time remains limited, and these trends should be interpreted with caution.

Another limitation stems from the study’s exclusive reliance on quantitative data collected through structured, closed-ended questions. The absence of open-ended responses or qualitative exploration limited our ability to capture deeper emotional reactions, personal motivations, or culturally situated narratives around vaccination. Future research could benefit from mixed-methods approaches that incorporate interviews or open-text responses to provide a richer understanding of public attitudes.

Additionally, the self-reported nature of the data makes it susceptible to common biases, such as social desirability bias (where participants provide answers they believe are socially acceptable) and recall bias (inaccurate recollection of past attitudes or behaviors). These factors may have affected both the consistency and reliability of responses, particularly in relation to attitudes toward Mpox and trust in public health communication (Polas, 2025; Efendić et al., 2022).

While the study compares two distinct waves of data collection, the cross-sectional design does not permit causal inference or the identification of intra-individual change over time. The observed trends in vaccine acceptance, perceived risk, and conspiracy beliefs are interpreted at the population level. Without panel data, we cannot determine whether the same individuals changed their attitudes between 2022 and 2025, or whether differences reflect variations in sample composition. Furthermore, the absence of qualitative components limits our insight into the emotional, cognitive, and social mechanisms that drive these changes. Future studies would benefit from longitudinal or mixed-methods designs that combine repeated survey data with in-depth interviews to more accurately capture the evolving nature of vaccine attitudes.

Taken together, these limitations underscore the need for cautious interpretation of our findings and highlight the importance of using more representative sampling techniques and multimodal data collection strategies in future research.

## 6. Conclusions

At the level of public perception, belief in the emergence of a new pandemic caused by Mpxv and in the likelihood of infection remains low. Although there has been a slight upward trend in public willingness to accept vaccination against Mpox during the analyzed period (2022–2025), overall acceptance remains limited. This is likely due to a combination of objective and subjective factors, including lingering negative public rhetoric surrounding vaccination from the COVID-19 pandemic.

Willingness to accept Mpox vaccination is influenced by key predictors such as age, gender, place of residence, and education level, though the strength of these associations varies across demographic groups.

These findings point to increasing polarization of public opinion on vaccination, with pro-vaccine and skeptical groups becoming more distinct based on belief systems and informational environments (Mønsted & Lehmann, 2022; Henkel et al., 2023; Jones & McDermott, 2022; Schmidt et al., 2018). This trend highlights the need for more carefully tailored communication strategies that account for the psychological and social factors shaping vaccine acceptance.

From a policy perspective, the findings offer preliminary insights that may help to guide future health communication efforts—while recognizing the limitations of a non-representative sample and the modest effect sizes observed. Although vaccine acceptance was significantly higher among individuals previously vaccinated against COVID-19 or through other voluntary programs, this association should be interpreted cautiously. Rather than implying direct causality, the results suggest that reinforcing existing positive attitudes through consistent, targeted messaging may be a productive approach.

The observed decline in conspiracy belief endorsement, and its reduced predictive power in 2025, may signal growing public resilience to misinformation—particularly when accurate information is sustained and accessible. However, these interpretations remain tentative and require further validation through broader, more representative studies. Public health authorities may still consider investing in media literacy and digital engagement initiatives, especially targeting vulnerable populations such as youth and individuals with lower levels of formal education. However, such programs should be guided by qualitative research and localized needs assessments to ensure contextual relevance.

Although lower levels of vaccine acceptance were observed among rural residents and women in this study, no causal evidence supports the design of specific interventions targeting these subgroups. Further research employing representative samples and experimental methods is needed to better understand the drivers of vaccine hesitancy within these populations. Nonetheless, community-based trust-building strategies—particularly those involving local intermediaries such as general practitioners or community leaders—remain promising pathways for engagement, pending further empirical validation.

## Figures and Tables

**Table 1:** Sociodemographic data of participants.

Variables		Year	
		2025 [%]	2022 [%]
Age (years)	18–25	17.01	30.44
	26–35	15.94	22.96
	36–45	40.04	21.25
	46–55	21.09	15.26
	56–65	5.05	7.11
	66+	0.87	2.98
	Total	100	100
Gender	Male	54.62	50.38
	Female	45.38	49.62
Residence	Urban	79.30	72.44
	Rural	20.70	27.56
Education Level	Pre-university	17.98	21.03
	University	82.02	78.97

**Table 2:** Comparative analysis of public perceptions on Mpox in 2022 and 2025: pandemic belief (Q1), fear of infection (Q2), and vaccination acceptance (Q3), with associated statistical tests.

Variable	Q1			Q2		
	2025	2022	Chi^2^	2025	2022	KS Stat
Age 18–25	19.6	31.3	95.05 (*p* < 0.01)	22.9	30.4	0.199 (*p* < 0.01)
Age 26–35	14.0	23.0	54.39 (*p* < 0.01)	20.1	33.4	0.254 (*p* < 0.01)
Age 36–45	9.0	15.4	48.73 (*p* < 0.01)	19.2	25.7	0.212 (*p* >0.05)
Age 46–55	9.2	9.8	36.79 (*p* < 0.01)	17.1	34.6	0.271 (*p* < 0.01)
Age 56–65	11.5	15.7	25.51 (*p* < 0.01)	11.5	36.1	0.212 (*p* > 0.05)
Age 66+	0.0	20.0	13.27 (*p* < 0.01)	0.0	20.0	0.347 (*p* < 0.01)
Male	7.8	19.0	159.52 (*p* < 0.01)	15.3	28.9	0.298 (*p* < 0.01)
Female	16.7	24.0	142.30 (*p* < 0.01)	23.3	33.5	0.211 (*p* < 0.01)
Urban	11.3	19.3	206.51 (*p* < 0.01)	18.6	31.5	0.252 (*p* < 0.01)
Rural	14.1	26.5	90.07 (*p* < 0.01)	20.2	28.9	0.258 (*p* < 0.01)
Pre-university	10.3	22.7	38.36 (*p* < 0.01)	17.3	30.8	0.241 (*p* >0.05)
University	12.2	20.9	195.08 (*p* < 0.01)	19.3	30.9	0.221 (*p* < 0.01)

Q1—Do you believe that monkeypox will turn into a pandemic? Q2—Are you afraid that you will contract Mpxv?

**Table 3:** Variable importance for vaccine acceptance prediction: 2022 vs. 2025.

Variable	Importance 2022	Importance 2025
Q4	0.976	0.779
Q5	0.005	0.152
Gender	0.004	0.040
Age	0.008	0.019
Education	0.002	0.006
Residence	0.004	0.004

Q4—Have you received optional vaccines? Q5—Have you been vaccinated against COVID-19?

**Table 4:** Comparative logistic regression of conspiracy predictors (2022 vs. 2025).

Misinformation Statement	Variable	Year	Coef	OR	CI 95% Low–High	*p*-Value	Φ 2022	Φ 2025
MS1	Residence	2022	0.66	1.95	1.30–2.92	<0.01	0.484	0.542
	Residence	2025	1.22	3.39	2.09–5.50	<0.01		
	Education		0.33	1.40	1.04–1.87	<0.05		
	Age		0.44	1.55	1.26–1.91	<0.01		
	Gender		−1.6	0.19	0.12–0.29	<0.01		
MS2	Residence	2022	−0.49	0.60	0.39–0.93	<0.05	0.187	0.251
	Education		−0.53	0.58	0.36–0.93	<0.05		
	Gender	2025	1.97	7.22	3.55–14.68	<0.05		
	Residence		−1.16	0.31	0.16–0.58	<0.01		
	Education		−0.43	0.64	0.43–0.95	<0.05		
MS3	Age	2022	0.15	1.17	1.02–1.34	<0.05	0.281	0.278
	Residence		−0.53	0.58	0.38–0.89	<0.05		
	Gender	2025	1.85	6.40	3.28–12.46	<0.01		
	Residence		−0.88	0.41	0.22–0.77	<0.01		
MS4	Residence	2022	−0.69	0.49	0.32–0.77	<0.01	0.244	0.270
	Education		−0.69	0.49	0.31–0.79	<0.01		
	Gender	2025	1.99	7.38	3.73–14.58	<0.01		
	Residence		−1.07	0.34	0.18–0.62	<0.01		
	Education		−0.41	0.65	0.44–0.96	<0.05		
MS5	Residence	2022	−0.55	0.57	0.37–0.87	<0.01	0.290	0.260
	Gender	2025	1.89	6.67	3.35–13.31	<0.01		
	Residence		−1.24	0.28	0.15–0.53	<0.01		
	Education		−0.50	0.60	0.40–0.89	<0.05		

MS1—Is monkeypox a real disease? MS2—Is there a global elite that spread monkeypox to reduce the world’s population? MS3—Did monkeypox emerge after the COVID-19 pandemic as a means of keeping the world population under control through fear? MS4—Are monkeypox vaccines designed to reduce the world’s population, similar to claims made about COVID-19 vaccines? MS5—Was monkeypox created because the population reduction from the COVID-19 pandemic was insufficient?

**Table 5:** Comparative logistic regression of conspiratorial beliefs (MS1–MS5) as predictors of Mpox vaccination intent (Q3) in 2022 and 2025.

Predictors	2022				2025			
	Coef.	OR	CI Low–High	*p*	Coef.	OR	CI Low–High	*p*
Monkeypox is real (MS1)	2.15	8.59	5.77–12.78	<0.01	−1.58	0.20	0.15–0.25	<0.01
Created by elites (MS2)	1.09	2.98	1.31–6.79	<0.01	−0.96	0.38	0.22–0.96	>0.05
Tool of manipulation (MS3)	−0.87	0.41	0.19–0.90	<0.05	0.899	2.45	1.15–4.91	<0.01
Vaccines for population reduction (MS4)	−0.51	0.59	0.24–1.41	>0.05	0.41	1.52	0.70–3.29	>0.05
Population control (MS5)	−0.94	0.38	0.15–0.96	<0.05	0.68	1.98	0.79–5.87	>0.05

## Data Availability

For the 2025 dataset, see https://figshare.com/articles/dataset/English_Dataset_mpox_2025_xlsx/29430269?file=55739684 (accessed on 10 August 2025). The 2022 dataset was previously published in an open-access article (Peptan et al., 2022). Its reuse in the present study complies fully with the CC BY license, and the dataset is included here strictly for comparative purposes with the newly collected 2025 data.

## References

[B1-behavsci-15-01175] Bachelet V., Adrián C., Navarrete M. S. (2022). Respondent-driven sampling: Advantages and disadvantages from a sampling method. Medwave.

[B2-behavsci-15-01175] Badea M. M., Mișacă L. C. (2021). Consultare publică privind măsurile COVID-19 luate de guvernul României în sectorul sănătății. Ars Aequi.

[B3-behavsci-15-01175] Bârgăoanu A. (2021). Dezordinea informațională—O criză care le exacerbează pe toate celelalte. Gândul.ro *(Blog)*.

[B4-behavsci-15-01175] Blănaru M. A., Vaida S. (2024). Systematic review: The relationship between vaccine hesitancy against COVID-19 and anxiety disorders. Studii și Cercetări.

[B5-behavsci-15-01175] Borcak D., Özdemir Y. E., Yesilbag Z., Ensaroğlu E., Akkaya S., Yaşar K. K. (2024). Assessment of knowledge and concern of people living with HIV regarding human Mpox and vaccination. Current HIV Research.

[B6-behavsci-15-01175] Cadeddu C. (2023). Vaccine hesitancy in Europe: The long and winding road. European Journal of Public Health.

[B7-behavsci-15-01175] Dascălu S., Geambașu O., Covaciu O., Chereches R. M., Diaconu G., Dumitra G. G., Gheorghita V., Popovici E. D. (2021). Prospects of COVID-19 vaccination in Romania: Challenges and potential solutions. Frontiers in Public Health.

[B8-behavsci-15-01175] Dobre M. (2021). Tipuri de argumentare greșită în perioada COVID-19. Revista de Filosofie.

[B9-behavsci-15-01175] Dutta S., Ghosh R., Dasgupta I., Sikdar P., Santra P., Maity D., Pritam M., Lee S. G. (2025). Monkeypox: A comprehensive review on mutation, transmission, pathophysiology, and therapeutics. International Immunopharmacology.

[B10-behavsci-15-01175] Efendić E., Olsen J., Schneider I., Anvari F., Arslan R. C., Elson M. (2022). Bias in self-reports: An initial elevation phenomenon. Social Psychological and Personality Science.

[B11-behavsci-15-01175] Emanuel E., Schaefer G., Leland R. J., Richardson H., Saenz C., Atuire C., Persad G. (2023). Equitable global allocation of monkeypox vaccines. Vaccine.

[B12-behavsci-15-01175] European Centre for Disease Prevention and Control (2025). Transmission of monkeypox virus clade I: Overall risk remains low in EU/EEA.

[B13-behavsci-15-01175] European Commission (2019). Vaccination: European Commission and World Health Organization join forces to promote the benefits of vaccines.

[B14-behavsci-15-01175] Gherheș V., Cernicova-Buca M., Fărcașiu M. A. (2023). Public engagement with Romanian government social media accounts during the COVID-19 pandemic. International Journal of Environmental Research and Public Health.

[B15-behavsci-15-01175] Graphs.ro (2025). Variola—Cazuri noi de variola maimuței pe zi în România.

[B16-behavsci-15-01175] Halder S. K., Sultana A., Himel M. K., Shil A. (2025). Monkeypox: Origin, transmission, clinical manifestations, prevention, and therapeutic options. Interdisciplinary Perspectives on Infectious Diseases.

[B17-behavsci-15-01175] Henkel L., Sprengholz P., Korn L., Betsch C., Böhm R. (2023). The association between vaccination status identification and societal polarization. Nature Human Behaviour.

[B18-behavsci-15-01175] Hong J., Pan B., Jiang H.-J., Zhang Q., Xu X., Jiang H., Ye J., Cui Y., Yan X., Zhai X., Yu Q. (2023). The willingness of Chinese healthcare workers to receive monkeypox vaccine and its independent predictors: A cross-sectional survey. Journal of Medical Virology.

[B19-behavsci-15-01175] Indiastari D., Fajar J. K., Tamara F., Runesi O., Hakim L. N., Chotimah K., Rahmani A., Saputro T. D., Afrilla D., Firmansyah E., Dau D., Dzhyvak V. (2024). Global prevalence and determinants associated with the acceptance of monkeypox vaccination. Narra Journal.

[B20-behavsci-15-01175] Jain N., Tanasov A., Chodnekar S. Y., Rakauskaitė A., Lansiaux E., Skuja S., Reinis A. (2022). Quantitative bibliometric excellence & productivity in monkeypox (Mpox) literature. Preprint.

[B21-behavsci-15-01175] Jentzsch A., Geier A. K., Bleckwenn M., Schrimpf A. (2022). Differences in demographics of vaccinees, access to, and satisfaction with SARS-CoV-2 vaccination procedures between German general practices and mass vaccination centers. Vaccines.

[B22-behavsci-15-01175] Jones D., McDermott M. (2022). The evolution and polarization of public opinion on vaccines. Public Opinion Quarterly.

[B23-behavsci-15-01175] Jung H.-S., Baek E., Park J.-Y., Hwang J.-H., Kwon S. (2024). Effects of perceived risk of COVID-19 on fear among visiting workers: Mediating role of perceived stress. Medicine.

[B24-behavsci-15-01175] Kanyo A., Dinu E., Rogozea L. (2021). Evaluarea nivelului de informare în mediu rural privind vaccinurile—Etapă esențială în realizarea unei campanii de prevenție. Jurnal Medical Brașovean.

[B25-behavsci-15-01175] Kirbiš A. (2023). The impact of socioeconomic status, perceived threat and healthism on vaccine hesitancy. Sustainability.

[B26-behavsci-15-01175] Lamot M., Kirbiš A. (2024). Multilevel analysis of COVID-19 vaccination intention: The moderating role of economic and cultural country characteristics. European Journal of Public Health.

[B27-behavsci-15-01175] Lawrence A. (2024). Assessing vaccine intentions, knowledge, self-efficacy, and trust: A cross-sectional study on perceptions of monkeypox vaccination and public health risk awareness in Makurdi, Benue State, Nigeria. Cureus.

[B28-behavsci-15-01175] León-Figueroa D. A., Barboza J. J., Siddiq A., Sah R., Valladares-Garrido M. J., Rodriguez-Morales A. J., Ahmed S. K. (2024). Knowledge and attitude towards Mpox: Systematic review and meta-analysis. PLoS ONE.

[B30-behavsci-15-01175] Lounis M., Hamimes A., Dahmani A. (2024). Assessment of monkeypox (Mpox) knowledge and vaccination intention among health and life sciences students in Algeria: A cross-sectional study. Infectious Disease Reports.

[B29-behavsci-15-01175] Lounis M., Riad A. (2023). Monkeypox (Mpox)-related knowledge and vaccination hesitancy in non-endemic countries: Concise literature review. Vaccines.

[B31-behavsci-15-01175] Mahameed H., Al-Mahzoum K., AlRaie L. A., Aburumman R., Al-Naimat H., Alhiary S., Barakat M., Al-Tammemi A. B., Salim N. A., Sallam M. (2023). Previous vaccination history and psychological factors as significant predictors of willingness to receive Mpox vaccination and a favourable attitude towards compulsory vaccination. Vaccines.

[B32-behavsci-15-01175] Matei E., Ilovan O. R., Sandu C. B., Dumitrache L., Istrate M., Jucu I. S., Gavrilidis A. A. (2021). Early COVID-19 pandemic impacts on society and environment in Romania: Perception among population with higher education. Environmental Engineering and Management Journal.

[B33-behavsci-15-01175] Mărcău F. C., Purec S., Niculescu G. (2022). Study on the refusal of vaccination against COVID-19 in Romania. Vaccines.

[B34-behavsci-15-01175] Moawad M. H. E., Taha A. M., Nguyen D., Ali M., Mohammed Y. A., Moawad W. A. E., Hamouda E., Bonilla-Aldana D. K., Rodriguez-Morales A. J. (2023). Attitudes towards receiving monkeypox vaccination: A systematic review and meta-analysis. Vaccines.

[B35-behavsci-15-01175] Mønsted B., Lehmann S. (2022). Characterizing polarization in online vaccine discourse—A large-scale study. PLoS ONE.

[B36-behavsci-15-01175] Ndembi N., Folayan M. O., Komakech A., Mercy K., Tessema S., Mbala Kingebeni P., Ngandu C., Ngongo N., Kaseya J., Abdool Karim S. S. (2025). Evolving epidemiology of Mpox in Africa in 2024. New England Journal of Medicine.

[B37-behavsci-15-01175] Oeser P., Grune J., Dedow J., Herrmann W. J. (2024). The 5C model and Mpox vaccination behavior in Germany: A cross-sectional survey. BMC Public Health.

[B38-behavsci-15-01175] Okutsu Y., Goromaru H. (2024). A study of the effect of COVID-19 on risk perception using qualitative time series data. 2024 IEEE/ACIS 9th International Conference on Big Data, Cloud Computing, and Data Science (BCD).

[B39-behavsci-15-01175] Omojunikanbi N. C. (2023). Public relations and effective communication during a global health crisis: Combating disinformation, misinformation, and fake news on COVID-19. International Journal of Communication and Health.

[B40-behavsci-15-01175] Ordeanu V. (2018). The strategic importance of vaccination for national defense and security. RJMM.

[B41-behavsci-15-01175] Peptan C., Băleanu V. D., Mărcău F. C. (2022). Study on the vaccination of the population of Romania against monkeypox in terms of medical security. Vaccines.

[B42-behavsci-15-01175] Peptan C., Mărcău F. C. (2024). Impactul informațiilor de tip fake news asupra problematicilor securitare.

[B43-behavsci-15-01175] Peptan C., Peptan S. E. (2021). Considerations regarding the security influences of the COVID-19 pandemic on the public health field in Romania. Journal of Research and Innovation for Sustainable Society.

[B44-behavsci-15-01175] Plummer R. (2022). Monkeypox: WHO declares highest alert over outbreak. BBC News.

[B45-behavsci-15-01175] Polas M. R. H. (2025). Common method bias in social and behavioral research: Strategic solutions for quantitative research in the doctoral research. Journal of Comprehensive Business and Administrative Research.

[B46-behavsci-15-01175] Rocha Y. M., de Moura G. A., Desidério G. A., de Oliveira C. H., Lourenço F. D., Nicolete L. D. d. F. (2021). The impact of fake news on social media and its influence on health during the COVID-19 pandemic: A systematic review. Journal of Public Health.

[B47-behavsci-15-01175] Sandu D. (2023). Social worlds of attitudes towards anti-COVID-19 vaccination: A multi-sited approach to contextualise a European society. Biomedical Science and Clinical Research.

[B48-behavsci-15-01175] Schmidt A. L., Zollo F., Scala A., Betsch C., Quattrociocchi W. (2018). Polarization of the vaccination debate on Facebook. Vaccine.

[B49-behavsci-15-01175] SHARE-ERIC (2021). Vaccination willingness in Europe: Who are the unvaccinated?.

[B50-behavsci-15-01175] Singh V., Dwivedi S., Agrawal R., Sadashiv, Fatima G., Abidi A. (2025). The human monkeypox virus and host immunity: Emerging diagnostic and therapeutic challenges. Infectious Disorders–Drug Targets.

[B51-behavsci-15-01175] Sîrbu D. (2020). The role of strategic communication in times of modern disinformation: Best practices and recommendations. International Scientific Conference STRATEGIES XXI: Strategic Changes in Security and International Relations.

[B52-behavsci-15-01175] Stoica Ș. E. (2024). Dinamica dezinformării. Impactul camerelor de ecou în modelarea opiniei publice online din România. Buletinul Universității Naționale de Apărare „Carol I”.

[B53-behavsci-15-01175] Sulaiman S. K., Tsiga-Ahmed F. I., Musa M. S., Makama B. T., Sulaiman A. K., Abdulaziz T. B. (2024). Global prevalence and correlates of Mpox vaccine acceptance and uptake: A systematic review and meta-analysis. Communications Medicine.

[B54-behavsci-15-01175] Székely I., Geambașu R., Kiss T., Toró T. (2024). Inherent attitudes or misplaced policies? Explaining COVID-19 vaccine hesitancy in Romania. East European Politics and Societies.

[B55-behavsci-15-01175] Tanashat M., Altobaishat O., Sharaf A., Moawad M. H. E. D., Al-Jafari M., Nashwan A. J. (2024). Assessment of the knowledge, attitude, and perception of the world’s population towards monkeypox and its vaccines: A systematic review and descriptive analysis of cross-sectional studies. Vaccine X.

[B56-behavsci-15-01175] Tian W., Zhao C., He J., Zhang G., Hu H., Du M., Zhao W., Ding N. (2024). How health anxiety is associated with perceived risk of reinfection among COVID-19 infected people after the epidemic control measures lifted in China: A multiple mediating and multi-group analysis. Psychology Research and Behavior Management.

[B57-behavsci-15-01175] Tiberiu I. (2019). Dimensiuni ale securității umane. Spre un model emergent de securitate individuală. Doctoral dissertation.

[B58-behavsci-15-01175] Tompea T. (2022). Comunicarea publică între informare și fake news în perioada pandemiei COVID-19. Revista Etică și Deontologie.

[B59-behavsci-15-01175] Trifunović V. (2022). Vaccine hesitancy in Western and Eastern Europe: The significance of contextual influences. Bulletin of the Institute of Ethnography.

[B60-behavsci-15-01175] Ungureanu D. (2022). Pandemia de COVID-19 în registrul vizual al mediilor de comunicare în masă din România: De la informare la anxietate. Cercetarea, dezvoltarea și inovația din perspectiva eticii globale.

[B61-behavsci-15-01175] Vâlcea C. S. (2023). Anti-science narratives as a form of legitimization of post-truth. Philologica Jassyensia.

[B62-behavsci-15-01175] Voinea C., Marin L., Vică C. (2023). The moral source of collective irrationality during COVID-19 vaccination campaigns. Philosophical Psychology.

[B63-behavsci-15-01175] Wang J., Fu L., Meng H., Wu K., Han B., Lin Y., Qi X. (2024). Knowledge, concerns, and vaccine acceptance related to Mpox (monkeypox) among university students in North and Northeast China: An online cross-sectional study. Human Vaccines & Immunotherapeutics.

[B64-behavsci-15-01175] Yappalparvi A., Gaidhane S., Padmapriya G., Kaur I., Lal M., Iqbal S., Prasad G. V. S., Pramanik A., Sharma P., Malik P., Vishwakarma T., Punia A., Jagga M., Mehta R., Sah S., Shabil M., Satapathy P., Bushi G., Parsa A. D., Kabir R. (2025). Prevalence of Mpox vaccine acceptance among students: A systematic review and meta-analysis. Vaccines.

[B65-behavsci-15-01175] Yatsuya H., Sasaki R., Ota A., Tabuchi T. (2022). Gender difference in fear and anxiety about and perceived susceptibility to COVID-19 in the third wave of pandemic among the Japanese general population: A nationwide web-based cross-sectional survey. International Journal of Environmental Research and Public Health.

